# Fitness trade-offs and the origins of endosymbiosis

**DOI:** 10.1371/journal.pbio.3002580

**Published:** 2024-04-12

**Authors:** Michael A. Brockhurst, Duncan D. Cameron, Andrew P. Beckerman

**Affiliations:** 1 Division of Evolution, Infection and Genomics, School of Biological Sciences, University of Manchester, Manchester, United Kingdom; 2 Department of Environmental and Earth Sciences, School of Natural Sciences, University of Manchester, Manchester, United Kingdom; 3 School of Biosciences, Ecology and Evolutionary Biology, University of Sheffield, Sheffield, United Kingdom; University of Texas Austin, UNITED STATES

## Abstract

Endosymbiosis drives evolutionary innovation and underpins the function of diverse ecosystems. The mechanistic origins of symbioses, however, remain unclear, in part because early evolutionary events are obscured by subsequent evolution and genetic drift. This Essay highlights how experimental studies of facultative, host-switched, and synthetic symbioses are revealing the important role of fitness trade-offs between within-host and free-living niches during the early-stage evolution of new symbiotic associations. The mutational targets underpinning such trade-offs are commonly regulatory genes, such that single mutations have major phenotypic effects on multiple traits, thus enabling and reinforcing the transition to a symbiotic lifestyle.

## Abstract 2

Endosymbiosis is common and has played an important role in the evolution of complex life, but its mechanistic origins remain unclear. This Essay explores the theory and experimental evidence for trade-offs in the early-stage evolution of endosymbiosis.

## Introduction

Endosymbiosis, wherein cells of one organism live within the cells (or organs) of another, has evolved many times across the tree of life, in a wide diversity of taxa, and commonly involves intimate interaction between organisms from different kingdoms of life [1]. By enabling species to gain entirely novel traits, such evolutionary mergers of formerly independent species have had a major role in evolutionary innovation [2]. Notable examples of symbiosis-mediated innovations include the gain of autotrophy [3] and gain of nitrogen fixation [4]. Such innovations have permitted symbiotic organisms to invade new ecological zones [5] and have led to the formation of entirely new biomes, such as coral reefs. Consequently, endosymbioses underpin the function of many varied ecosystems, spanning terrestrial, freshwater and marine habitats [6]. By opening up new ecological opportunities, endosymbioses can thus act as key innovations that, over evolutionary timescales, can catalyze diversification and fuel adaptive radiation [7–9], although not always [10].

Besides their role in biodiversity, endosymbioses have also enabled the evolution of more complex organisms [11] by allowing the compartmentalization of functions into specialized structures or organs, thereby increasing organismal multifunctionality and modularity [12]. Most fundamentally, this is evident in the symbiotic origin of the organelles of eukaryotic cells, which perform specialized metabolic functions that would be inefficient (or impossible) if performed in the bulk cytoplasm. Such improved efficiency is believed to have provided the necessary energetic potential for the evolution of larger, multicellular organisms [13]. While eukaryotic organelles are an extreme example, the benefits of compartmentalization are evident more generally across diverse endosymbioses [12].

Despite the ecological and evolutionary importance of endosymbioses, their origins remain poorly understood and difficult to explain. Although many extant endosymbioses appear mutually beneficial, this need not have been the case at their origin [14]. Evolutionary theory suggests that explaining the origin of endosymbiosis through mutualism is challenging because it not only requires that symbiosis be beneficial for both parties, but also that the fitness interests of these unrelated species be aligned, such that whatever increases the fitness of one partner necessarily benefits the other [15]. These conditions are unlikely to be met in newly established partnerships. Accordingly, theory suggests that mutual benefit may be an improbable mechanism for explaining the origin of endosymbiosis [15]. Exploitation, on the other hand, offers a potentially more plausible route for the formation of endosymbiosis [16], and either the host or the endosymbiont can engage in exploitation of their partner. In this Essay, we focus principally on the situation where hosts exploit beneficial endosymbionts. Here, the host benefits at the symbiont’s expense and, provided hosts exert sufficient control over the beneficial symbionts to enforce interaction, the origin of endosymbioses can then be explained entirely from the self-interest of the host species. Once established, and seemingly regardless of the mode of origin (i.e., by mutualism or exploitation by hosts), hosts then often establish a degree of vertical transmission (such that endosymbionts are passed from mother to daughter at cell division) or mechanisms of endosymbiont selection (such that noncooperative endosymbionts can be excluded). This increased fidelity between symbiotic partners sets the scene for the evolution of dependence, whereby adaptation to the symbiotic state is associated with the loss of free-living (Box 1) ability [17]. The particular combination of vertical transmission and dependence necessarily creates strong alignment of the fitness interests of symbiotic partners [18].

**Box 1. Glossary**

**Free-living**

Where potentially symbiotic species live without their symbiotic partner species.

**Pseudogenes**

Genes that have accumulated deleterious mutations to an extent that they no longer produce functional proteins.

**Genetic drift**

A nonselective process leading to change in mutation frequencies.

**Facultative symbiosis**

Where one or both partners in a symbiosis retain the ability to adopt a free-living lifestyle.

**Negative genetic correlation**

Where fitness of a genotype is negatively correlated between 2 or more contrasting ecological niches.

**Conditionally deleterious mutation**

A mutation that is selectively neutral in a focal niche but is deleterious to fitness in alternative niche(s).

**Obligate symbiosis**

Where both species in a symbiosis are no longer capable of living without their symbiotic partner.

**Positive genetic correlation**

Where fitness of a genotype is positively correlated between 2 or more contrasting ecological niches.

The purpose of this Essay is to explore the evolutionary processes driving such adaptation to the symbiotic state and the concomitant loss of free-living fitness, which ultimately leads to increased dependence and alignment of the fitness interests of symbiotic partners.

## Conceptualizing endosymbiosis as niche adaptation

Obligate endosymbionts often have highly reduced genomes, far smaller than those of free-living relatives, encoding fewer functions and containing higher numbers of pseudogenes (Box 1) [19]. As such, the evolution of obligate endosymbionts has typically been viewed through a lens of genome degradation: a progressive loss of function driven by random mutations accumulating due to genetic drift (Box 1) within small populations that are strictly clonal [20]. While genome degradation is likely to be a major part of the story of obligate endosymbiont evolution, the same is often not the case for facultative symbionts (Box 1) or those without strict vertical transmission, such as Rhizobia, which commonly retain comparatively large genomes. Nonetheless, the prevailing view that progressive genome degradation driven by genetic drift predominates endosymbiont evolution misses potentially important selection-driven evolutionary processes occurring earlier during the establishment of symbiosis. This limited perspective likely arises because most of what we know of endosymbiont evolution comes from studies of long-established interactions, where signals of earlier evolutionary events are likely to have been lost amid the noise of subsequent genetic drift.

What might these selection-driven evolutionary processes be and what kinds of signals would they leave behind in endosymbiont populations? For endosymbionts, the transition to endosymbiosis is one commonly involving a shift from an exterior free-living to an interior within-host environment, which is conceptually equivalent to what any population experiences when colonizing a new ecological niche. As such, to predict the potential evolutionary pathways for endosymbionts upon forming a symbiosis, we can borrow from the body of theory exploring evolutionary diversification and ecological specialization [21,22]. Formally, ecological specialists display a negative genetic correlation (Box 1) across ecological niches, such that adaptation to one niche is associated with reduced fitness in an alternative, contrasting niche, termed a cost of adaptation [23]. Crucially, negative genetic correlations between fitness in free-living and within-host niches due to costs of adaptation can build up through distinct genetic mechanisms, with different consequences for the rate and repeatability of evolutionary trajectories. Below, we place the origin of endosymbiosis within this evolutionary diversification framework.

It is highly probable that the new within-host niche of nascent endosymbionts will vary from their ancestral free-living niche along multiple axes. Key axes include environmental conditions, resources, and external threats. For example, pH and osmolarity are likely to be key environmental variables; within-host environments are likely to be less variable than exterior environments, and these factors may be at very different levels between free-living and within-host niches. The identity and variability of nutrients and energy sources (for example, organic versus inorganic nitrogen) will also differ between free-living and within-host environments. Furthermore, the presence, identity, variability, and virulence of biotic threats (including predators, viruses, and phages) will also differ between free-living and within-host niches; although, within-host niches are not without threats, notably the host immune response. Consequently, if sufficiently contrasting, these free-living and within-host niches are likely to impose divergent selection, favoring distinct sets of traits and adaptations in each. If we assume that the ancestor was well-adapted to the original exterior environment, then adaptation to the within-host niche is unlikely to improve free-living fitness, but may reduce it if there is a cost of adaptation [23]. Such a cost of adaptation would arise when a positive direct response to selection in terms of improved within-host fitness is accompanied by a negative indirect response to selection in terms of reduced free-living fitness. This causes a negative genetic correlation between fitnesses in the free-living and within-host niches.

**Fig 1:**
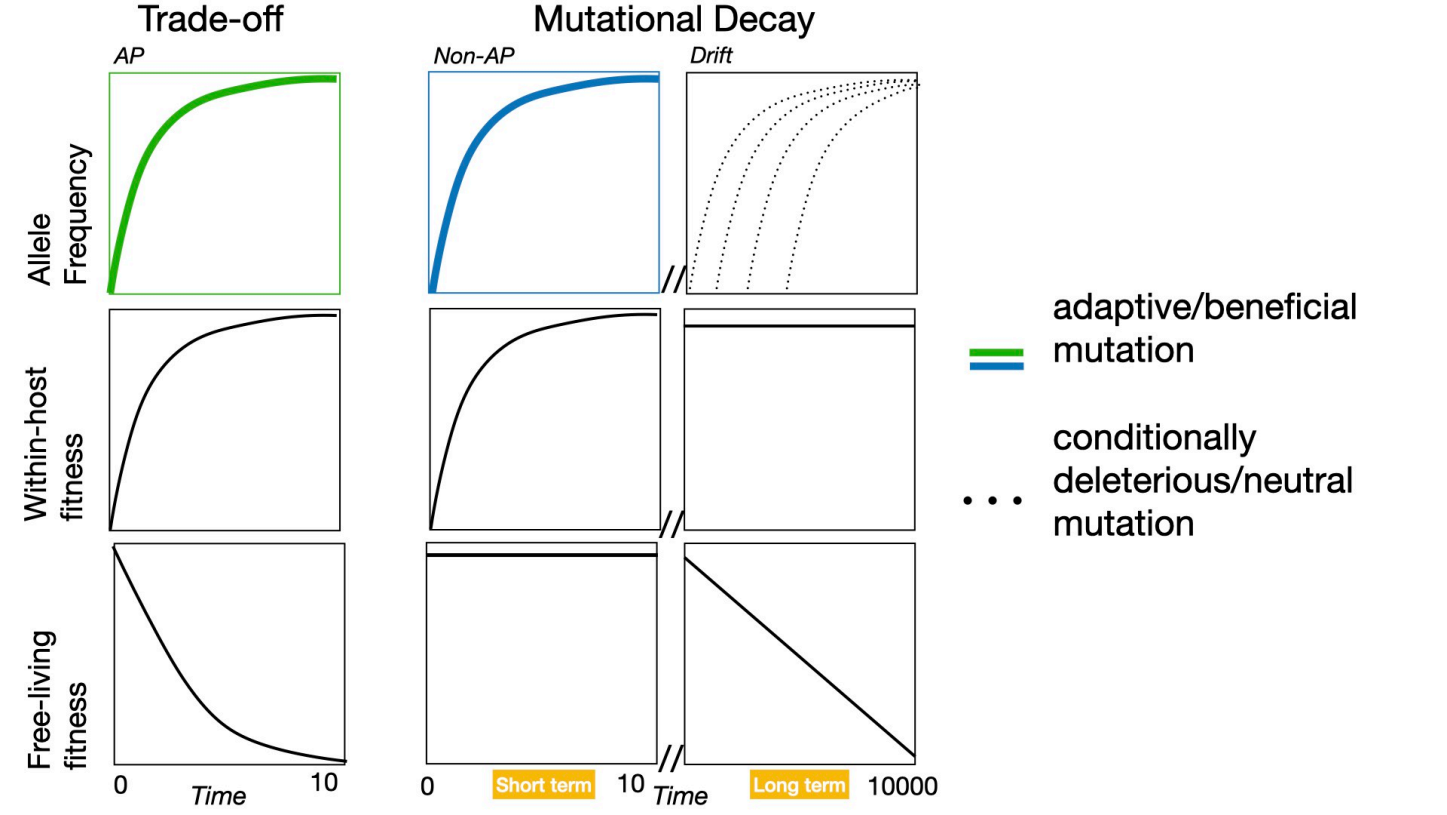
Trade-offs versus mutational decay in the evolution of endosymbiosis. Under trade-offs, beneficial mutations (green line) in the intracellular niche cause reduced fitness in the extracellular niche through antagonistic pleiotropy (AP). Under mutational decay, beneficial mutations (blue line) in the intracellular niche have no effect on extracellular fitness (non-AP), and negative genetic correlations build up over longer timescales though accumulation by genetic drift of (nearly) neutral mutations that are conditionally deleterious in the extracellular niche (dotted line).

A cost of adaptation can arise through 2 principle genetic mechanisms: mutational decay or a trade-off arising from antagonistic pleiotropy (Fig 1) [23]. The mechanism most commonly implicated in the evolution of symbioses, as explained above, is mutational decay. Here, the new endosymbiont adaptations selected in the within-host niche themselves have no detrimental effect on free-living fitness, but other traits crucial for free-living undergo mutational decay owing to disuse. Mutations can accumulate in the genes encoding such traits, which although (nearly) neutral in the within-host niche, cause progressive loss of fitness in the free-living niche. Such conditionally deleterious mutations (Box 1) can become fixed by genetic drift alone and are not mechanistically linked to the selective process driving endosymbiont within-host adaptation. Alternatively, the adaptive mutations that improve endosymbiont fitness in the within-host niche could themselves directly reduce fitness in the free-living niche, termed antagonistic pleiotropy. Here, endosymbiont within-host adaptation is mechanistically linked to the loss of free-living fitness, and the consequent negative genetic correlation is known as a trade-off.

Both mutational decay and antagonistic pleiotropy thus create negative genetic correlations among non-symbiotic and endosymbiotic genotype fitnesses across free-living and within-host niches. However, we would expect the dynamics and repeatability of the evolutionary processes driving these negative genetic correlations to differ (Fig 1). Evolution driven by selection on a trade-off should happen much faster than mutational decay by genetic drift. As such, trade-offs would be expected to cause rapid loss of extracellular fitness strongly reinforcing symbiosis, compared to the more progressive and gradual fitness decline caused by mutational decay. Moreover, the order in which mutations are fixed should be more predictable for selection on trade-offs, sequenced according to their fitness effects in the within-host niche, whereas (nearly) neutral mutations may become fixed in any order. By extension, we would expect that selection-driven evolution would result in more repeatable evolutionary outcomes between independent populations (i.e., parallel evolution), but that the outcomes of mutational decay would be closer to random sampling of mutational events per population. However, deviations from randomness, such as mutational biases, and/or mild selective benefits to losing free-living traits (for example, owing to their metabolic cost) could enhance the predictability of mutational decay.

## Evidence for the role of fitness trade-offs in symbiosis

**Fig 2:**
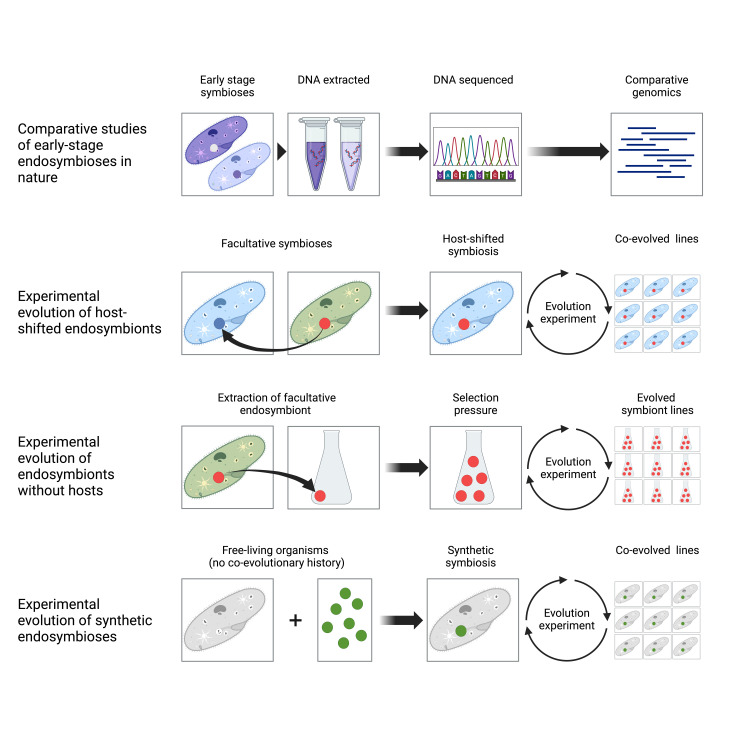
Experimental approaches to studying the role of trade-offs in symbiosis. There are 4 main experimental approaches to studying the role of trade-offs in the evolution of symbiosis: comparative studies of early-stage endosymbioses in nature; experimental evolution of host-shifted endosymbiosis; experimental evolution of endosymbionts without hosts; and experimental evolution of synthetic endosymbioses. The most powerful approach for understanding the origins of symbiosis is to experimentally evolve newly established synthetic symbioses because this directly recapitulates an origin event that is usually lost deep in evolutionary time for most extant symbioses. Created using Biorender.

The dichotomy we outline between trade-offs and mutational decay is, of course, an oversimplification. Indeed, it is probable that both processes combine to determine the evolution of endosymbionts. Nonetheless, a clear expectation deriving from their comparison is that selective processes driven by trade-offs are likely to dominate the early-stage evolution of endosymbioses, whereas mutational decay will become increasingly dominant in later-stage, longer-established endosymbioses. As such, it is likely that traditional approaches for studying endosymbiont evolution (for example, by comparing genomes of long-established endosymbionts with distant free-living relatives) are unlikely to be able to distinguish between these mechanisms. In this section, we examine the experimental approaches that enable us to study this question (Fig 2) and what data already exist to support a role for trade-offs in the evolution of endosymbiosis.

### Comparative studies of early-stage endosymbioses in nature

In rare cases, a nascent endosymbiosis in nature has been caught in the act of adapting to its new within-host niche, enabling direct observation of early-stage evolutionary events. For example, a comparison of 25 genomes of the bacterial insect endosymbiont *Arsenophonus*, spanning free-living environmental, facultative endosymbiotic, and obligate endosymbiotic (Box 1) lifestyles, revealed that the initial transition from the exterior environment to the within-host niche was associated with multiple acquisitions of mobile genetic elements, including both plasmids and prophages, that carried novel functions for host interactions (e.g., type-3 secretion system effectors) [24]. This drove substantial genome expansion that was facilitated by loss of functional CRISPR-Cas systems, which would otherwise have defended the bacterial genome against phages and other mobile genetic element DNA. Together, these data suggest a trade-off, wherein CRISPR-Cas systems are beneficial in the free-living niche (e.g., by defending against foreign DNA and phage infection), but become costly for endosymbionts adapting to the within-host niche, where rapid evolutionary innovation driven by horizontal gene transfer is required. Furthermore, it is probable that these loss-of-function mutations in the CRISPR-Cas systems will exhibit antagonistic pleiotropy because of higher phage infection pressure in the free-living niche. After this early-stage evolution, obligately endosymbiotic *Arsenophonus* follows a well-worn path shared by other insect endosymbionts, such as Sodalis, of mutational decay and genome reduction [25]. Increasing the diversity of symbiotic systems and broadening the range of host interaction types included in comparative analyses (e.g., to include facultative and parasitic intermediates) is likely to open up opportunities to discover other such endosymbiotic transitions in action.

### Experimental evolution of host-shifted endosymbionts

While some facultative endosymbionts, like *Arsenophonus*, can represent a relatively earlier stage in the evolution of symbiosis than obligate associations, an added benefit of studying facultative endosymbioses is that they are more experimentally tractable than obligate associations. This is because the symbiotic partners tend to be amenable to independent culturing, enabling experimenters to create new host–symbiont combinations that do not exist in nature and follow their evolution. In particular, facultative endosymbionts that would naturally have a free-living stage can be serially passaged within hosts for multiple generations, selecting for adaptation to the within-host environment and experimentally recapitulating a transition to obligate endosymbiosis. However, only a handful of such studies have tested for changes in free-living fitness following experimental evolution of host-switched endosymbionts to test for trade-offs. Several studies report rapid host adaptation by bioluminescent *Vibrio fischeri*. Here, following passaging in novel squid hosts, *V. fischeri* showed altered levels of bioluminescence matching the preference of their new host (which varies among host taxa) [26,27]. Genome sequencing of evolved lines and reverse genetics revealed that host adaptation was due to mutations in a single regulatory gene, *binK*, which controls both biofilm production and quorum sensing regulators of bioluminescence [28]. *binK* mutants had higher fitness within hosts, owing to an altered bioluminescence level and enhanced colonization and immune evasion abilities, but suffered reduced fitness in free-living non-host environments, demonstrating a trade-off. A clear limitation of the host-shift approach is that the endosymbionts are likely to already be adapted to some components of the within-host environment, and as such this approach may well miss some of the key early-stage adaptations.

### Experimental evolution of endosymbionts without hosts

An alternative approach to host-switching experiments is to instead experimentally evolve endosymbionts without hosts in a free-living-like environment. This experimental design effectively reverses the process that originally occurred during the transition to endosymbiosis, selecting for adaptation to the exterior environment and thus potentially revealing trade-offs between fitness in free-living and within-host niches. Perhaps surprisingly, given that this experimental design is arguably simpler than host-switching, we found few examples of beneficial endosymbionts for which this has been attempted (although multiple examples exist for pathogens showing loss of host adaptation in free-living adapted lines, e.g., [29]). In one study, 9 symbiotic *Bradyrhizobium* strains were experimentally evolved in lab media for 30 growth cycles, showing that 4/9 strains adapted to lab conditions, and that for 2 of these, this increased free-living fitness was associated with reduced symbiotic performance in host plants [30]. In another study, free-living populations of bioluminescent *V. fischeri* were evolved at different pH levels and then tested their fitness in both free-living and within-host niches [31]. While populations that were evolved in high, low, or fluctuating pH environments gained higher free-living fitness, only those that evolved in high pH showed reduced within-host fitness (i.e., negative genetic correlation), whereas those that evolved in low or fluctuating pH showed higher within-host fitness (i.e., positive genetic correlation; Box 1). Unfortunately, distinguishing between trade-off versus mutational decay mechanisms was not possible in either of these studies because no whole-genome sequencing analysis was performed, but both studies highlight the potential of no-host evolution experiments to deconstruct free-living niches to understand key environmental axes potentially driving trade-offs.

### Experimental evolution of synthetic endosymbiosis

A key limitation of host-shift experiments is that different symbiotic host environments will often be more similar to each other than to non-symbiotic exterior environments, and thus between-host contrasts may be less likely to elicit trade-offs. Moreover, neither host-switching nor no-host selection of endosymbionts directly examine the transition from free-living to endosymbiosis. Overcoming these limitations are evolution experiments that select originally non-symbiotic organisms to become de novo symbionts. Such experiments are extremely challenging, not least because these synthetic symbioses are liable to be highly unstable and tend to have very low initial growth rates. Nonetheless, synthetic symbioses are exceptionally powerful tools in that they truly recapitulate the evolutionary events and processes of the formation of a new symbiosis in a controlled experiment. Surprisingly, experiments creating synthetic endosymbioses have a long history, dating back more than 50 years [32], but only more recently have these kinds of studies identified the underlying adaptive genetic mechanisms and potential trade-offs involved.

Conversion of various bacterial taxa into “synthetic rhizobia” has been achieved by transferring the plasmids encoding the symbiosis genes from nitrogen-fixing rhizobia symbionts [33]. Experimental evolution of the plant pathogen *Ralstonia solanacearum* carrying a symbiotic plasmid from rhizobium *Cuprividus taiwanensis* led to it becoming a nodule-forming symbiont of *Mimosa pudica* [34]. This adaptation to legume symbiosis involved loss of virulence pathways and the type-3 secretion system, as well as remodeling of quorum-sensing regulation, all of which reduced pathogenicity while improving nodulation and infection success [33], which together is likely to prevent subsequent reversion to the ancestral pathogenic lifestyle. In an even more remarkable example, the obligate bacterial symbiont of stinkbugs was replaced with lab *Escherichia coli* [35]. Following 2 years of passaging through insects, an initially unstable and low fitness association evolved into a beneficial symbiosis, wherein the host now required *E. coli* for its growth and maturation. Although not intracellular, the *E. coli* did become localized to the specialized symbiotic organ and was vertically transmitted to eggs. Evolved *E. coli* had acquired mutations in regulators of carbon catabolite repression (CCR), *cyaA* or *crp*, down-regulating hundreds of genes involved in carbon metabolism. Reconstruction of single mutations in either of these regulatory genes in the ancestral *E. coli* was sufficient to transform the ancestor into a beneficial symbiont. Although much improved as symbionts, the evolved *E. coli* had impaired free-living growth, reduced capsule production, and smaller cell size than their ancestor, indicating strong antagonistic pleiotropic effects of the CCR mutations and a clear fitness trade-off between symbiotic and free-living lifestyles. Regulatory mutations have also been implicated in the emergence of host-beneficial symbiotic lineages of the soil bacterium *Pseudomonas lurida* experimentally evolved in the guts of nematode worm hosts [36]. Notably, these regulatory mutations improved symbiotic fitness at the expense of reduced fitness in the exterior free-living environment, again indicating an important role for antagonistic pleiotropy.

## Conclusion and future outlook

Trade-offs are likely to have an important role in the origin of endosymbiosis. Only trade-offs cause rapid loss of free-living fitness through antagonistic pleiotropy as an endosymbiont adapts to its new within-host niche, enforcing the endosymbiotic lifestyle. By contrast, under mutational decay, loss of free-living fitness occurs more gradually, such that nascent endosymbionts could inhabit either niche for a more extended period. These mechanisms are not, however, mutually exclusive and it is likely that early evolutionary events mediated by trade-offs become hidden by subsequent mutational decay in many extant symbioses. This limits our ability to study the role of trade-offs in symbiosis retrospectively, although some systems such as the bacterium *Arsenophonus* do offer glimpses of the entire transition from free-living to obligate endosymbiont [24].

Instead, experimental evolutionary biologists have found multiple creative ways to study trade-offs prospectively, including studying free-living adaptation, host-shifted associations, and creating entirely synthetic symbioses (Fig 2). Of these approaches, synthetic symbioses offer the greatest potential to study symbiotic origins, because these directly recapitulate this event, which is lost deep in evolutionary time for most natural symbioses. Common mutational targets causing antagonistic pleiotropic trade-offs are regulatory genes, including quorum-sensing systems and global metabolic regulators. Here, a single mutation can have major phenotypic effects through altering the expression of many other genes, exemplified by the *E. coli* synthetic endosymbionts of stinkbugs, in which mutation of the CCR regulator altered expression of hundreds of genes, remodeling metabolism and altering cell shape [35]. Wholesale changes in the expression of the genome will be likely to relax selection on regions no longer expressed in the within-host niche, enabling their degradation through mutational decay over longer timescales. The apparent importance of regulatory mutations in trade-offs highlights the need to integrate multiple omics data—including transcriptomes, proteomes, and metabolomes—to fully understand how these levels of cellular organization interconnect and manifest as an integrated endosymbiotic phenotype.
